# SAP deletion promotes malignant insulinoma progression by inducing CXCL12 secretion from CAFs via the CXCR4/p38/ERK signalling pathway

**DOI:** 10.1111/jcmm.18397

**Published:** 2024-05-20

**Authors:** Guangchun Jiang, Shuo Xu, Xiaobin Mai, Juan Tu, Le Wang, Lijing Wang, Yaping Zhan, Yan Wang, Qianqian Zhang, Lingyun Zheng, Jiangchao Li, Pei Tang, Cuiling Qi

**Affiliations:** ^1^ School of Basic Medical Sciences Guangdong Pharmaceutical University Guangzhou Guangdong China

**Keywords:** CAF, CXCL12, insulinoma growth, insulinoma metastasis, malignant insulinoma, SAP

## Abstract

Malignant insulinoma is an extremely rare type of functioning pancreatic neuroendocrine tumour with a high degree of malignancy and a high incidence of metastasis. However, it is still unclear how malignant insulinomas develop and metastasize. Serum amyloid P component (SAP), a member of the pentraxin protein family, is an acute‐phase protein secreted by liver cells. The role of SAP in insulinoma and the related mechanism are still unknown. To determine the effect of SAP on insulinoma, we crossed Rip1‐Tag2 mice, which spontaneously develop insulinoma, and SAP knockout (KO) mice to generate Rip1‐Tag2;SAP^−/−^ mice. We found that SAP deletion significantly promoted the growth, invasion and metastasis of malignant insulinoma through C‐X‐C motif chemokine ligand 12 (CXCL12) secreted by cancer‐associated fibroblasts (CAFs). Further study showed that SAP deletion promoted CXCL12 secretion by CAFs through the CXCR4/p38/ERK signalling pathway. These findings reveal a novel role and mechanism of SAP in malignant insulinoma and provide direct evidence that SAP may be a therapeutic agent for this disease.

## 1 INTRODUCTION

Insulinoma, an islet β‐cell tumour, is the most common pancreatic neuroendocrine tumour (pNET), with an annual incidence of one to five per million population.^1^ Most insulinomas are benign, and between 2.4% and 17.9% of insulinomas are malignant, with an average of 8.4%. Malignant insulinoma has often already metastasized at the time of initial diagnosis, most commonly to local lymph nodes and the liver.^2^ The medical treatment of malignant insulinoma includes surgical excision, chemotherapy, tumour‐targeted radiotherapy and treatment with somatostatin analogues. However, these therapies are unable to cure patients with malignant insulinoma. Thus, the development of novel therapies based on the current knowledge of tumour biology is warranted.

Serum amyloid P component (SAP) is a member of the pentraxin (PTX) protein family, which includes short‐chain PTXs, such as SAP and C‐reactive protein (CRP), and long‐chain PTXs, such as PTX3.^3^ CRP and SAP are acute‐phase reactive proteins. CRP is a major acute‐phase protein in humans, while SAP is an acute‐phase protein in mice.^4^ SAP is produced primarily in the liver and is secreted into the blood by hepatocytes and has a distinctive flattened β‐jelly roll structure and calcium‐dependent ligand binding activity.^5^ SAP plays an important role in the innate immune system by interacting with complement system components and binding to the Fcγ receptor.^6^ In addition, SAP reduces neutrophil activation and recruitment, regulates macrophage activation and inhibits fibroblast differentiation.^7^, ^8^, ^9^, ^10^ It has been reported that SAP has significant effects on autoimmune disease,^11^ dermal wound healing,^12^ cardiac and pulmonary fibrosis,^3^ allergic airway disease,^13^ kidney injury,^14^ radiation‐induced oral mucositis,^15^ and so on. It was previously reported that the treatment with recombinant human SAP protein improved the lung function of patients with pulmonary fibrosis in a phase 1B clinical trial.^16^ Furthermore, it has been reported that the serum SAP concentration in patients with breast cancer is related to breast cancer progression, while the serum SAP concentration exhibits no difference in patients with colon cancer.^17^ However, whether SAP plays a role in malignant insulinoma progression is not yet known.

Chemokines have important effects on the progression of malignant tumours. C‐X‐C motif chemokine ligand 12 (CXCL12) is reportedly secreted by cancer‐associated fibroblasts (CAFs).^18^ CAFs, which generally originate from activated fibroblasts within the tumour microenvironment, overexpress a specific subset of biomarkers, such as α‐SMA, FAP‐α and PDGFR‐α/β, according to the tumour type.^19^ CAFs generally secrete cytokines and chemokines, such as CXCL12, TGF‐β and TNFα.^20^ CXCL12 plays a crucial role in tumour growth and metastasis by binding to its receptor, CXCR4, in the tumour cell membrane.^21^ CXCL12/CXCR4 signalling has also been shown to activate several signalling pathways, including the mitogen‐activated protein kinase (MAPK)/ERK and phosphatidylinositol 3‐kinase/Akt pathways, in various cell lines.^22^ Thus, activation of the CXCL12/CXCR4/MAPK pathway is important for tumour growth and metastasis. We investigated the relationship between SAP‐silenced CAFs and activation of the CXCL12/CXCR4/p38/ERK pathway in insulinoma.

In the present study, we investigated the roles of SAP in malignant insulinoma using the Rip1‐Tag2 mouse model, in which mice spontaneously develop multiple insulinomas because the SV40 large T‐antigen (Tag) is expressed in pancreatic β cells. In Rip1‐Tag2 mice, normal islets gradually develop into hyperplastic, dysplastic islets and angiogenic islets by approximately 6 weeks of age. Tumours appear in Rip1‐Tag2 mice at 9–10 weeks of age. The tumours in Rip1‐Tag2 mice are classified as noninvasive tumours (T), invasive carcinoma type I (IC1; focal regions of invasion) or invasive carcinoma type II (IC2; widespread invasion).^23^ Here, we found that SAP deletion promoted CXCL12 secretion by CAFs, which activated the CXCR4/p38/ERK signalling pathway, thereby promoting insulinoma growth, invasion and metastasis in Rip1‐Tag2 mice. Importantly, SAP may be a novel therapeutic agent for malignant insulinoma.

## 2 MATERIALS AND METHODS

### 2.1 Mice

Rip1‐Tag2 transgenic mice, serum amyloid P component knockout (SAP^−/−^) mice and SAP transgenic (SAP‐Tg) mice were obtained from Professor Geng and their phenotypes were identified by PCR using the following primers: Rip1‐Tag2 mice: 5′‐GGACAAACCACAACTAGA ATG‐3′ (forward) and 5′‐CAGA GC AG AA TT GT G GAGTGG‐3′ (reverse); SAP^−/−^ mice: 5′‐TACAG TGACC TTTCC CGCT CT CA GA GTCT‐3′ (forward), 5′‐ACGACTCACT ATAGGGCGAATTGGGTACAC‐3′ (reverse). Male Rip1‐Tag2 mice were crossed with female SAP^−/−^ mice or SAP‐Tg mice to generate Rip1‐Tag2;SAP^−/−^ mice or Rip1‐Tag2;SAP‐Tg mice. The animals were housed and used in experiments in accordance with institutional guidelines. All animal procedures were approved by the Medical Research Animal Ethics Committee of Guangdong Pharmaceutical University [No. gdpulacspf2021005].

### 2.2 Murine model of spontaneous insulinoma

Rip1‐Tag2, Rip1‐Tag2; SAP^−/−^ and Rip1‐Tag2; SAP‐Tg mice were characterized as previously described.^24^ Angiogenic islets and tumours in Rip1‐Tag2 and Rip1‐Tag2; SAP^−/−^ mice were evaluated as previously described.^25^ The length and width of the tumours were measured using a Vernier calliper, and the tumour volume was calculated with the following formula: 0.52 × length × width^2^. For the survival assay, the mice were kept in a pathogen‐free environment and the survival times of the Rip1‐Tag2, Rip1‐Tag2; SAP^−/−^ and Rip1‐Tag2; SAP‐Tg mice were recorded.

### 2.3 Histology

The pancreases were harvested from Rip1‐Tag2 and Rip1‐Tag2;SAP^−/−^ mice and immediately fixed with 4% paraformaldehyde overnight. The next day, the pancreases were embedded in paraffin and sectioned at a thickness of 3 μm. The tissues were stained with haematoxylin and eosin, analysed and classified as noninvasive tumour (T), IC1 or IC2 tissues as previously described. The number of T, IC1 and IC2 was counted in the pancreatic tissues of Rip1‐Tag2 and Rip1‐Tag2;SAP^−/−^ mice and expressed as the percentage of the total number of T, IC1 and IC2 per the pancreatic tissues. The data were independently collected and evaluated by two experimenters using a double‐blind protocol.

### 2.4 Evaluation of tumour metastasis

The livers, lungs, spleens, kidneys and intestines of 12‐week‐old Rip1‐Tag2 and Rip1‐Tag2;SAP^−/−^ mice were isolated and fixed with 4% paraformaldehyde. Next, they were dehydrated, embedded in paraffin, serially sectioned and stained with haematoxylin and eosin. To assess the incidence of tumour metastasis, two experienced pathologists examined the haematoxylin and eosin‐stained sections and evaluated the percentage of mice with metastatic foci using a double‐blind protocol.

### 2.5 Immunohistochemical and immunofluorescent staining

Immunohistochemical (IHC) and immunofluorescent (IF) of pancreatic tissues were performed as previously described.^26^ Briefly, pancreatic tissue slides were dewaxed, hydrated and boiled in citrate buffer. Next, endogenous peroxidase was quenched using a H_2_O_2_‐methanol solution at 37°C for 30 min, and the sections were blocked with 10% BSA. The slides were incubated with a rabbit anti‐Ki67 polyclonal antibody (cat. no. ab279653, Abcam, Cambridge, CB, UK), a rabbit anti‐CXCL12 antibody (cat. no. ab25117, Abcam), a mouse anti‐α‐SMA antibody (cat. no. ABM0052, Abbkine, Wuhan, China) overnight at 4°C. The binding of the primary antibodies to the tissues was evaluated using HRP‐conjugated secondary antibodies plus DAB or the appropriate fluorescently labelled secondary antibodies. All sections were the counterstained with haematoxylin or 4′,6‐diamidino‐2‐phenylindole. Images were acquired at 400 × magnification, and the number of Ki67‐positive (Ki67^+^) cells was determined and expressed as the percentage of the total number of cells per field. The fields were randomly selected from at least three tumours per mouse (typically 10–15) for four to five mice per group. The data were independently collected and evaluated by two researchers (Guangchun Jiang and Cuiling Qi) using a double‐blind protocol.

### 2.6 Protein chip assay

Mouse protein chip assay kit containing 43 different inflammatory factors was purchased from RayBiotech (cat. no. AAM‐INF‐1, RayBiotech, Guangzhou, China). The protein chip assay was performed as described in the manufacturer's instructions. Briefly, sera from Rip1‐Tag2 and Rip1‐Tag2;SAP^−/−^ mice were obtained and incubated with antibodies of the protein chip arrays overnight at 4°C. Next, the membranes were treated with a biotinylated antibody cocktail after being washed to remove unbound proteins and this step was followed by incubation with horseradish peroxidase‐streptavidin. The arrays were analysed and protein abundances were quantified using the RayBiotech Q‐analyser Tool (RayBiotech).

### 2.7 Cell lines and cell culture

NIT‐1 cells (mouse insulinoma β cells), RIN‐m5F cells (rat insulinoma β cells) and L929 cells (mouse fibroblasts) were purchased from Shanghai Biochemical Cell Research Institute. All the cells were cultured in RPMI 1640 medium (Gibco, Carlsbad, CA, USA) supplemented with 10% FBS and 1% penicillin/streptomycin (cat. no. 15140122, Gibco, Carlsbad, CA, USA) at 37°C with 5% CO_2_. All cell lines used in the experiments were routinely tested for mycoplasma contamination and were confirmed to be mycoplasma‐free.

### 2.8 Conditioned medium

NIT‐1 cells were cultured to 40%–50% confluence in complete medium in Petri dishes, after which the complete medium was replaced with RPMI 1640 medium containing 10% FBS. To obtain the conditioned medium (CM), after 48 h of culture, the cell culture supernatant was collected and centrifuged at 1500 r/min for 5 min to remove cell debris. The CM was stored at −20°C for subsequent experiments. To generate CAFs, L929 cells were treated with NIT‐1‐derived CM. Briefly, the CM was added to complete growth medium at a ratio of 1:1 and L929 cells were stimulated for 72 h at 37°C. Then, CM from the CAFs was collected in the same manner.

### 2.9 Transfection

To transfect SAP siRNA (RiboBio, Guangzhou, Guangdong, China) into L929 cells, the cells were treated with Lipofectamine 3000 (cat. no. 18324020, Thermo Fisher Scientific, CA, USA) according to the manufacturer's guidelines. Briefly, after 12 h, the transfection medium was replaced with complete medium. Protein was extracted from the collected cells after 48 h.

### 2.10 Enzyme‐linked immunosorbent assay

The serum of Rip1‐Tag2 and Rip1‐Tag2;SAP^−/−^ mice and culture supernatant from NFs and CAFs with or without SAP siRNA transfection were collected. Subsequently, the CXCL12 concentrations in the mouse serum samples and cell supernatants were measured using a Mouse CXCL12 enzyme‐linked immunosorbent assay (ELISA) Kit (cat. no. RX202859M, RuiXin Biotech, Quanzhou, China) according to the manufacturer's instructions.

### 2.11 Cell migration and invasion assays

Cell migration and invasion assays were performed as previously described using a Boyden chamber with a polycarbonate filter (Transwell 24, 8 μm pore, Costar, Cambridge, MA, USA) coated with (cell invasion assay) or without (cell migration assay) Matrigel (cat. no. 356234, BD Biosciences San Jose, CA, USA). A total of 8 × 10^4^ NIT‐1 cells was resuspended in 200 μL of serum‐free RPMI 1640 medium and then seeded into the upper chambers on top of the membranes coated with or without Matrigel. PBS, CXCL12 (50 ng/mL), SAP (300 ng/mL) or CAFs were added to the bottom chamber. After 16 h (migration assay) or 36 h (invasion assay), the NIT‐1 cells remaining in the chamber were removed by scraping using a cotton swab. The migrated or invaded cells on the lower surface of the chamber membrane were fixed with 4% paraformaldehyde for 30 min and stained with 0.1% crystal violet for 30 min. The migrated or invaded cells were counted in at least six randomly selected fields per membrane at 400 × magnification. The experiments were performed in triplicate.

### 2.12 Western blotting

Cells were harvested for protein extraction. The protein concentration was quantified using the BCA method. Equal amounts of total protein were separated by 10%–12% SDS‐PAGE and electroblotted onto polyvinylidene difluoride membranes (cat. no. IPVH00010, Millipore, Billerica, MA, USA). Subsequently, the membranes were blocked with 5% nonfat dry milk for 1 h at room temperature. The membranes were then incubated with primary antibodies at 4°C overnight prior to incubation with HRP‐conjugated secondary antibodies for 1 h at room temperature. Signals were detected by the ECL method. The following primary antibodies were used: anti‐SAP (cat. no. GTX55792, Gene Tex, CA, USA), anti‐α‐SAM (cat. no. ab5694, Abcam, Cambridge, CB, UK), anti‐CXCR4 (cat. no. sc‐9046, Santa Cruz Biotechnology, TX, USA), anti‐p38 (cat. 8690S, Cell Signaling Technology, Danvers, MA, USA), anti‐phospho‐p38 (cat. no. 4511S, Cell Signaling Technology, Danvers, MA, USA), anti‐ERK (cat. no. 9102S, Cell Signaling Technology, Danvers, MA, USA), anti‐phospho‐ERK (cat. no.9101S, Cell Signaling Technology, Danvers, MA, USA), and anti‐GAPDH (cat. no. 2118S, Cell Signaling Technology, Danvers, MA, USA).

### 2.13 Statistical analysis

All the statistical analyses were performed using GraphPad Prism–9.0 software (GraphPad Software, CA). Comparisons between two groups were performed using Student's *t*‐test. Differences among multiple groups were evaluated using one‐way ANOVA followed by the Bonferroni correction. A *p*‐value of <0.05 or <0.01 was considered statistically significant or very statistically significant respectively.

## 3 RESULTS

### 3.1 SAP deficiency promotes insulinoma growth

**FIGURE 1:**
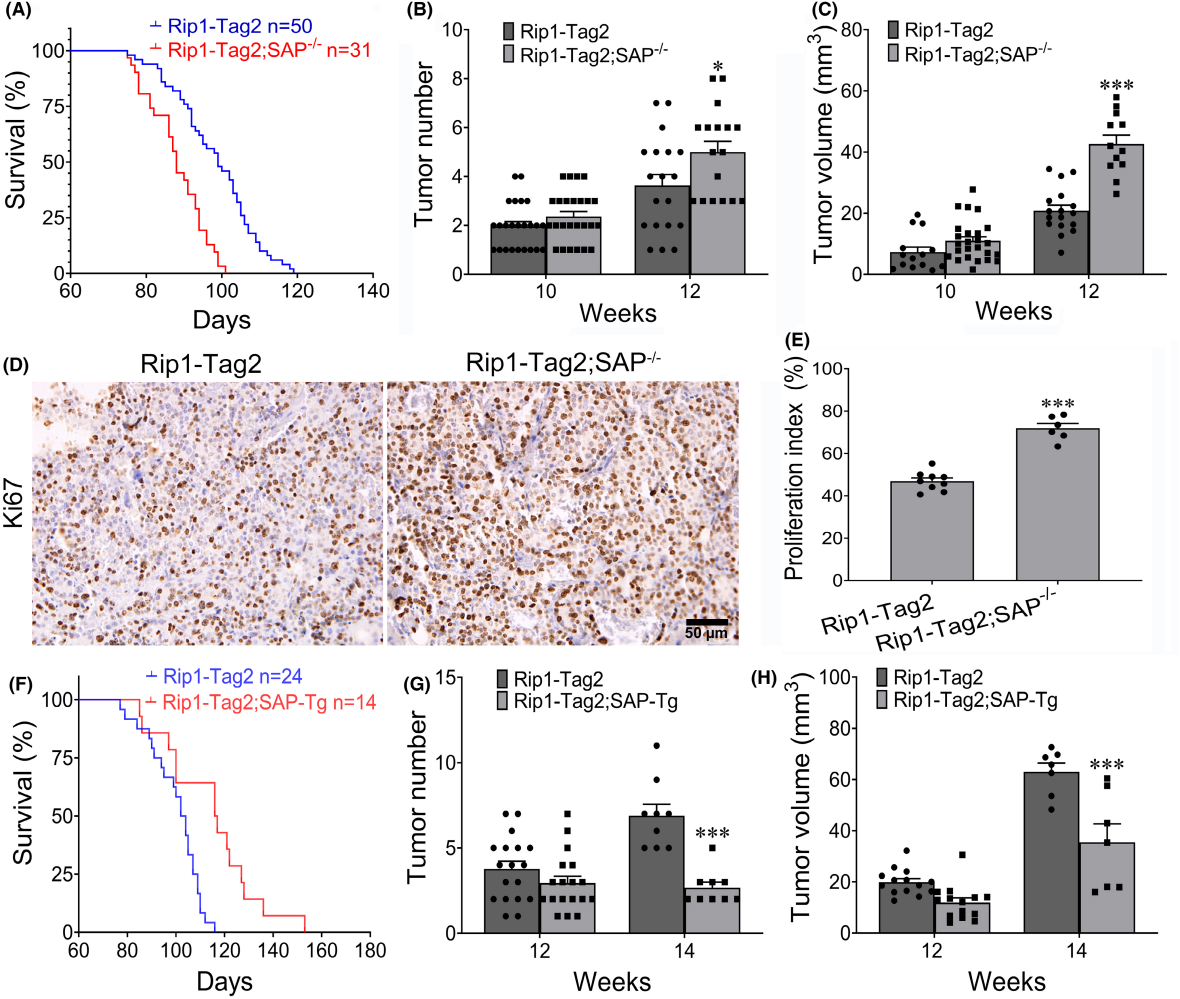
The effect of serum amyloid P component (SAP) on insulinoma growth. (A) The survival times of Rip1‐Tag2 and Rip1‐Tag2;SAP^−/−^ mice. (B, C) Tumour numbers (B) and tumour volumes (C) in Rip1‐Tag2 and Rip1‐Tag2;SAP^−/−^ mice. (D, E) Representative immunohistochemical images of insulinomas from Rip1‐Tag2 and Rip1‐Tag2;SAP^−/−^ mice. Statistical analysis demonstrated that SAP deletion promoted tumour cell proliferation. The results are representative of at least four sections per mouse from a minimum of three mice per group. (F) The survival times of Rip1‐Tag2 and Rip1‐Tag2;SAP‐Tg mice. (G, H) Tumour numbers (G) and tumour volumes (H) in Rip1‐Tag2 and Rip1‐Tag2;SAP‐Tg mice. * *p* < 0.05; *** *p* < 0.001. Scale bar = 50 μm.

To explore the effect of SAP on insulinoma growth in Rip1‐Tag2 mice, we crossed SAP knockout mice with Rip1‐Tag2 mice to generate Rip1‐Tag2;SAP^−/−^ mice. SAP deletion significantly decreased the survival time of Rip1‐Tag2;SAP^−/−^ mice (Figure 1A). Compared with their Rip1‐Tag2 counterparts, Rip1‐Tag2;SAP^−/−^ mice also had more tumours and larger tumour volumes at the age of 12 weeks (Figure 1B,C). However, there were no significant differences in the tumour volume or tumour number between 10‐week‐old Rip1‐Tag2 mice and their Rip1‐Tag2;SAP^−/−^ counterparts (Figure 1B,C). To further determine the effect of SAP deletion on insulinoma cell proliferation, immunohistochemical staining for Ki67 was performed on the tumour tissues of Rip1‐Tag2 and Rig1‐Tag2;SAP^−/−^ mice. The results showed that the proliferative capacity of insulinoma cells in the tumour tissues of Rig1‐Tag2;SAP^−/−^ mice was significantly greater than that in the tumour tissues of Rip1‐Tag2 mice (Figure 1D,E). These results show that SAP deletion significantly promotes insulinoma growth. To further determine the effect of SAP on insulinoma growth, we crossed the SAP overexpression (SAP‐Tg) mice with Rip1‐Tag2 mice to establish the Rip1‐Tag2;SAP‐Tg mice. We found that SAP overexpression significantly increased the survival time of Rip1‐Tag2 mice (Figure 1F). Compared with their Rip1‐Tag2 counterparts, Rip1‐Tag2;SAP‐Tg mice also had few tumours and smaller tumour volumes at the age of 14 weeks (Figure 1G,H). However, there no significant difference of tumour number and tumour volume between 12‐week‐old Rip1‐Tag2 mice and Rip1‐Tag2;SAP‐Tg mice. These results show that SAP overexpression significantly inhibits malignant insulinoma growth.

To further determine our findings in mice, we investigated the SAP expression in the tumour tissues from clinic. We found that the expression level of SAP in the tumour tissues from the patients with malignant insulinoma was significantly decreased than that in the normal islet (Figure S1A,B). Meanwhile, the serum level of SAP in Rip1‐Tag2 mice was significantly decreased compared with that in C57BL/6J mice (Figure S1C). SAP deletion significantly decreased SAP concentration in the serum and liver tissues of Rip1‐Tag2 mice (Figure S1D).

### 3.2 SAP deletion promotes insulinoma invasion in Rip1‐Tag2 mice

**FIGURE 2:**
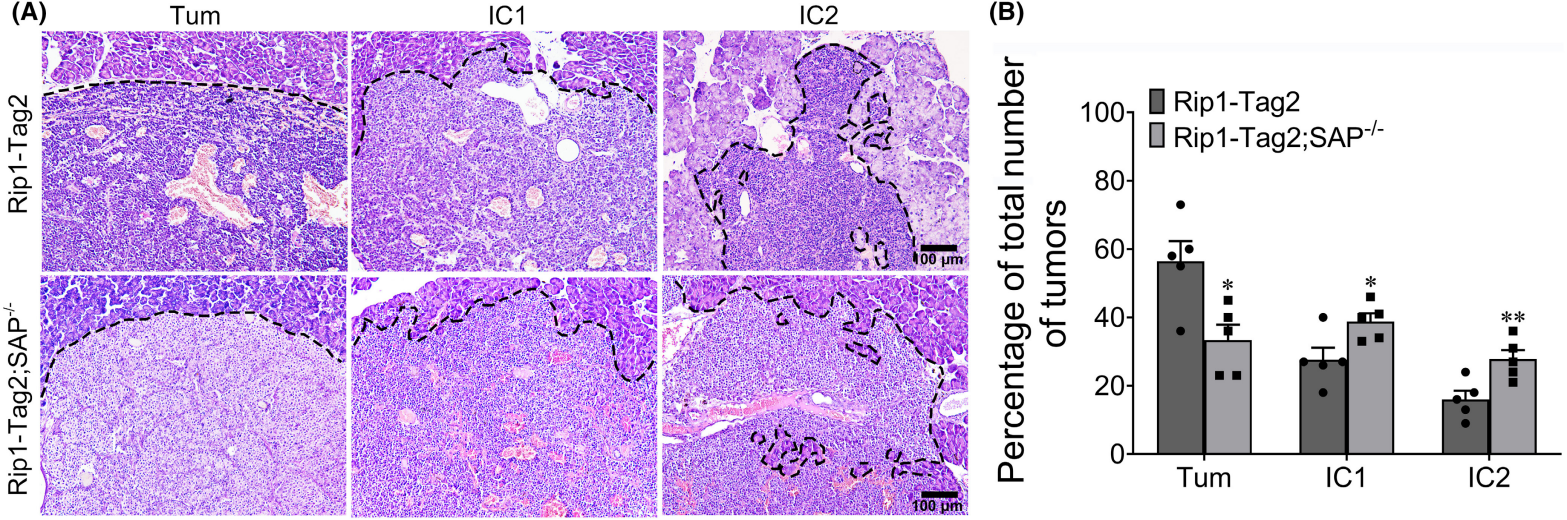
Serum amyloid P component (SAP) deficiency promotes tumour invasion. (A) Haematoxylin and eosin staining of a noninvasive insulinoma, a focally invasive IC1 insulinoma, and a broadly invasive IC2 insulinoma from a Rip1‐Tag2 mouse and a Rip1‐Tag2;SAP^−/−^ mouse. (B) Quantification of tumour invasiveness, shown as the percentages of noninvasive tumours, IC1 tumours, and IC2 tumours in Rip1‐Tag2 and Rip1‐Tag2;SAP^−/−^ mice at 12 weeks of age. * *p* < 0.05; ** *p* < 0.01. Scale bar = 100 μm.

To further determine the role of SAP deletion in insulinoma progression, we examined the distribution of the distinctive invasive phenotypes in Rip1‐Tag2 mice and Rip1‐Tag2;SAP^−/−^ mice. The overall distributions of both the focally invasive IC1 tumours and the widely invasive IC2 tumours in Rip1‐Tag2;SAP^−/−^ mice were significantly greater than those in Rip1‐Tag2 mice (Figure 2A,B). These results suggest that SAP deficiency increases the development of invasive carcinoma lesions.

### 3.3 SAP deletion promotes insulinoma metastasis

**FIGURE 3:**
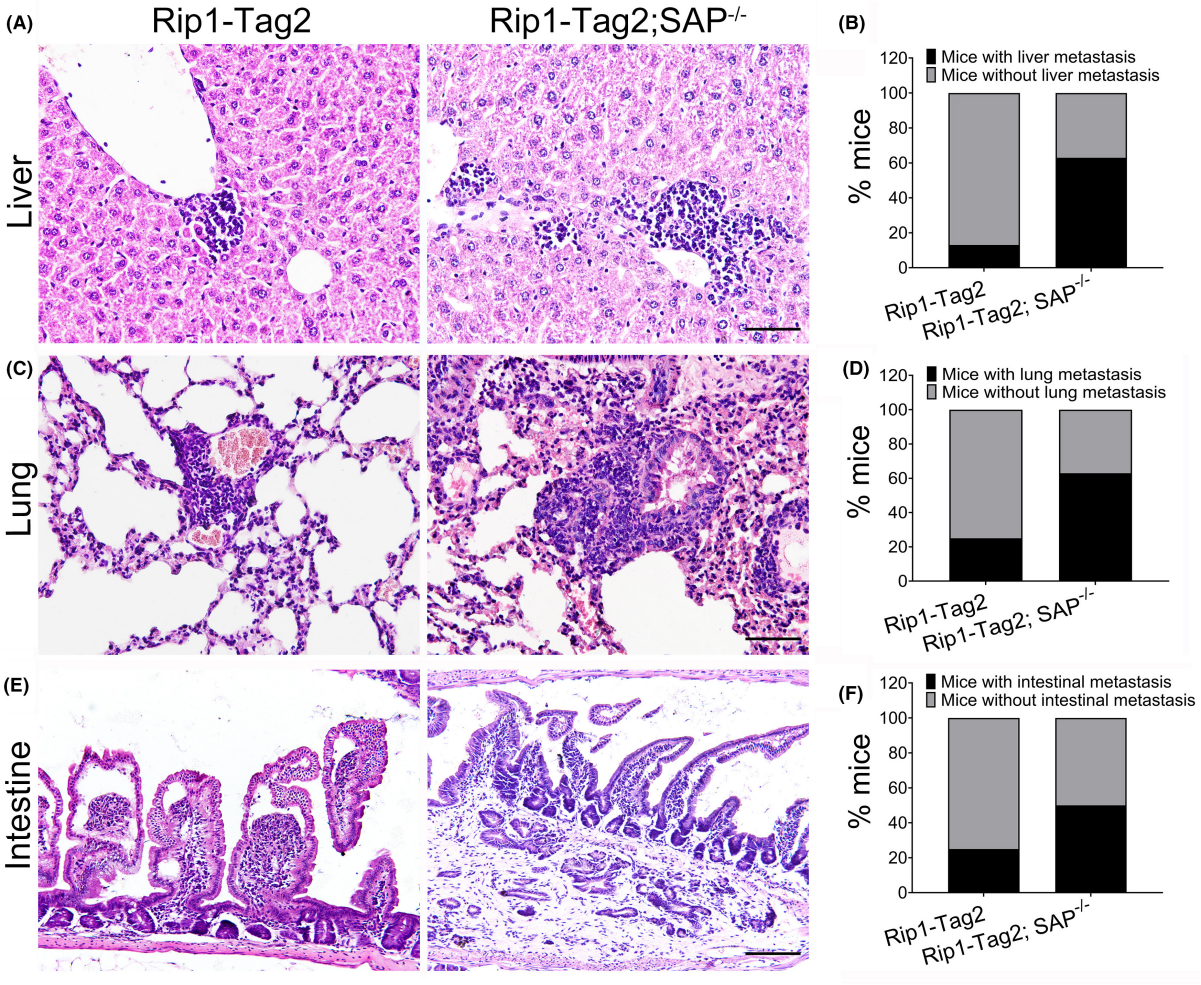
Serum amyloid P component (SAP) deficiency promotes insulinoma metastasis. (A, C, E) Representative haematoxylin and eosin staining images of the livers, lungs and intestines from Rip1‐Tag2 mice and Rip1‐Tag2; SAP^−/−^ mice. (B, D, F) The percentages of mice with liver, lung and intestinal metastasis were calculated. Scale bar = 50 μm.

Given that SAP deletion is capable of promoting insulinoma growth and invasion, SAP deletion may play an important role in insulinoma metastasis. To investigate the effect of SAP deletion on insulinoma metastasis, the livers, lungs and intestines of Rip1‐Tag2 mice and Rip1‐Tag2;SAP^−/−^ mice were harvested and stained with haematoxylin and eosin. As expected, we found that there were large numbers of metastatic tumour foci in the livers, lungs and intestines of Rip1‐Tag2;SAP^−/−^ mice (Figure 3A,C,E). The percentage of Rip1‐Tag2;SAP^−/−^ mice with liver, lung and intestinal metastasis was significantly increased compared with that of Rip1‐Tag2 mice at the age of 12 weeks (Figure 3B,D,F). Our results indicate that SAP deletion promotes insulinoma metastasis.

### 3.4 SAP deletion increases the concentration of CXCL12 secreted by CAFs

**FIGURE 4:**
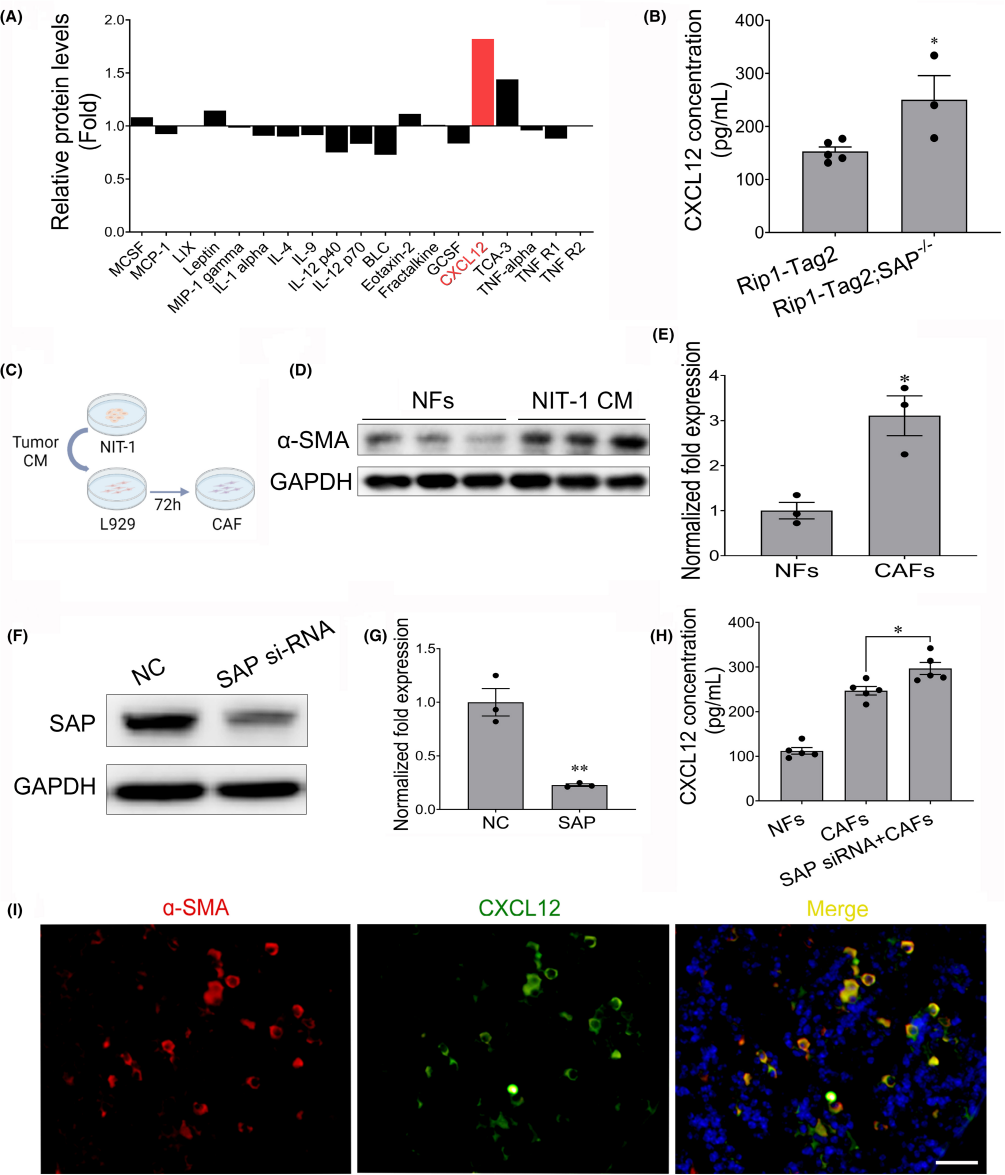
Serum amyloid P component (SAP) deletion increases the amount of CXCL12 secreted by CAFs. (A) The protein chip array data demonstrated that the CXCL12 concentration was increased in the serum of Rip1‐Tag2;SAP^−/−^ mice compared with the serum of Rip1‐Tag2 mice. (B) Serum concentration of CXCL12 in Rip1‐Tag2 mice and Rip1‐Tag2;SAP^−/−^ mice. (C) The stably transduced cell lines were processed through the indicated workflow. (D, E) The protein expression of α‐SMA was increased after L929 cells were treated with CM derived from NIT‐1 cells for 72 h. (F, G) SAP RNA interference significantly inhibited SAP protein expression in CAFs. (H) SAP knockdown significantly increased the CXCL12 concentration in CAF culture medium. (I) Representative immunofluorescent images of CXCL12 and α‐SMA for CAFs in the insulinoma tissues from Rip1‐Tag2 mice. CXCL12 expressed in α‐SMA positive cells in the tumour tissues of Rip1‐Tag2. Scale bar = 25 μm. * p < 0.05; ** *p* < 0.01.

To investigate how SAP deficiency promotes insulinoma progression, a protein chip array analysis was performed to detect changes in the concentrations of inflammation‐related proteins in the serum of Rip1‐Tag2 mice and Rip1‐Tag2;SAP^−/−^ mice. The CXCL12 concentration was found to be increased approximately two fold in the serum of Rip1‐Tag2;SAP^−/−^ mice compared with the serum of Rip1‐Tag2 mice (Figure 4A). To further determine whether CXCL12 is upregulated, we performed an ELISA and found that the concentration of CXCL12 in the serum of Rip1‐Tag2;SAP^−/−^ mice was greater than that in the serum of Rip1‐Tag2 mice (Figure 4B). Some studies have reported that CAFs can secrete CXCL12, which plays an important role in tumour progression.^27^, ^28^, ^29^ Then, to transform normal L929 mouse fibroblasts into CAFs, we treated L929 cells with CM derived from NIT‐1 cells for 72 h (Figure 4C) and found that the protein expression level of α‐SMA, a CAF‐specific marker, was significantly increased (Figure 4D,E). SAP knockdown decreased SAP expression in CAFs (Figure 4F,G). Furthermore, the secretion of CXCL12 was greater in the CAF culture medium than in the L929 normal fibroblast culture medium (Figure 4H). Interestingly, the secretion of CXCL12 was significantly increased after SAP expression was silenced (Figure 4H). To further determine that CXCL12 was secreted by CAFs, immunofluorescent staining of CXCL12 and α‐SMA for CAFs was investigated. We observed that CXCL12 expressed in α‐SMA positive cells in the tumour tissues of Rip1‐Tag2 (Figure 4I). Furthermore, the SAP concentration was very low in the serum and liver tissues of Rip1‐Tag2;SAP^−/−^ mice (Figure S1C,D). These results showed that SAP deletion increased the secretion of CXCL12 by CAFs.

### 3.5 SAP knockdown promotes the migration and invasion of NIT‐1 cells through CXCL12 secreted by CAFs

**FIGURE 5:**
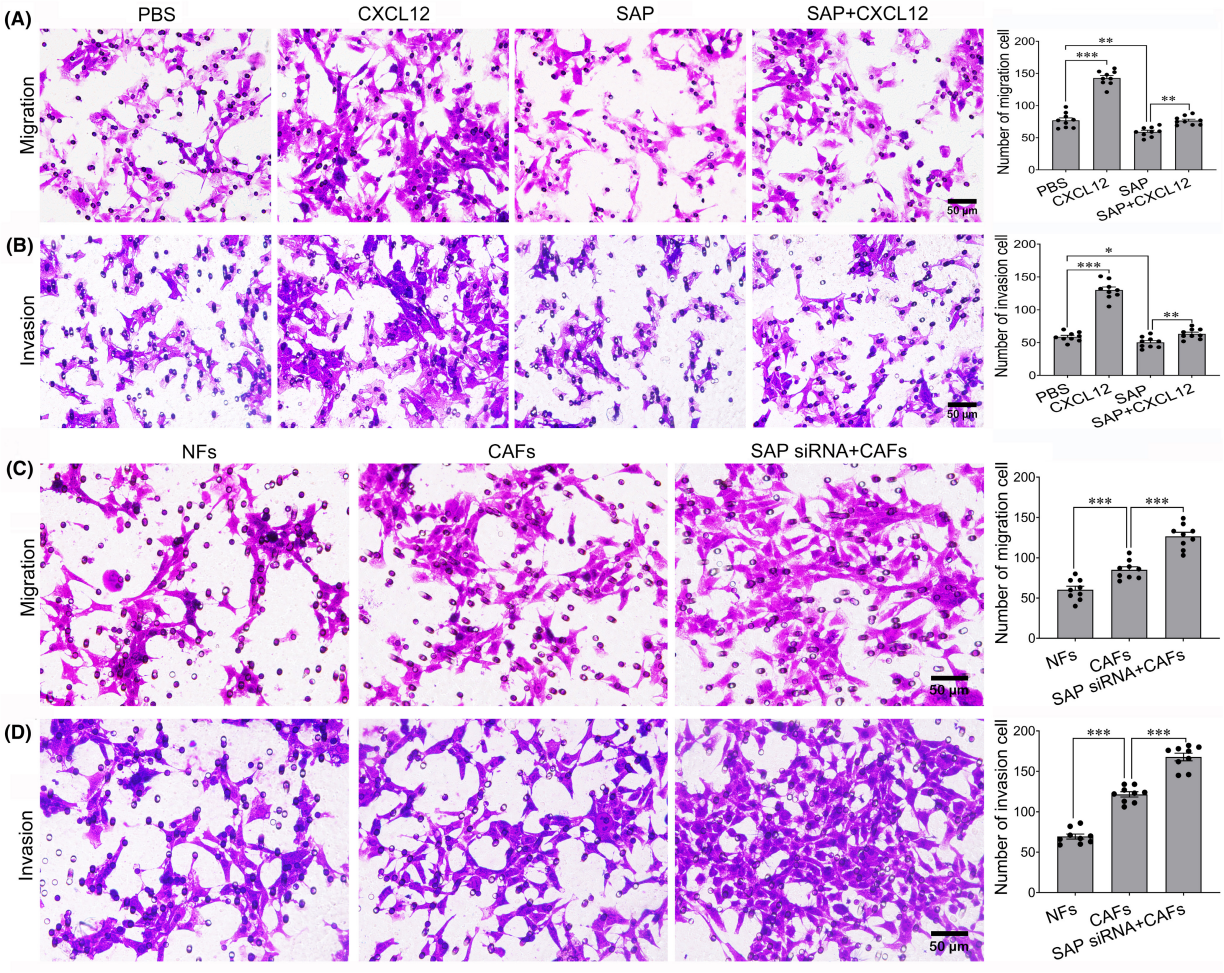
Effect of SAP knockdown on NIT‐1 cell migration and invasion through CAF‐derived CXCL12. (A) Representative images of migrated NIT‐1 cells. CXCL12 significantly promoted NIT‐1 cell migration. Furthermore, the ability of NIT‐1 cells treated with CXCL12 to migrate was reduced after serum amyloid P component (SAP) treatment. (B) Representative images of invaded NIT‐1 cells. CXCL12 significantly promoted NIT‐1 cell invasion. Furthermore, the ability of NIT‐1 cells treated with CXCL12 to invade was reduced after SAP treatment. (C) Representative images of migrated NIT‐1 cells. The ability of NIT‐1 cells to migrate was significantly increased after SAP was silenced. (D) Representative images of invaded NIT‐1 cells. The ability of NIT‐1 cells to invade was significantly increased after SAP was silenced. * *p <* 0.05; ** *p <* 0.01; *** *p <* 0.001. Scale bar = 50 μm.

To determine whether CXCL12 affects the migration and invasion of NIT‐1 cells in vitro, Transwell cell migration and Matrigel invasion assays were performed. Compared with PBS‐treated control NIT‐1 cells, CXCL12‐stimulated NIT‐1 cells showed significantly increased migratory and invasive capacities (Figure 5A,B). The numbers of migrated and invaded cells treated with SAP were significantly lower than those of the corresponding control cells (Figure 5A,B). Furthermore, the migratory and invasive capacities of NIT‐1 cells were decreased by SAP treatment after CXCL12 stimulation. To further investigate whether SAP affects the migratory and invasive capacities of NIT‐1 cells through CXCL12 secreted by CAFs, NIT‐1 cells were cocultured with CAFs with SAP silencing. The results showed that the migratory and invasive capacities of NIT‐1 cells were increased after coculture with CAFs, while SAP knockdown in CAFs further promoted NIT‐1 cell migration and invasion (Figure 5C,D). These results indicate that SAP knockdown in CAFs promotes NIT‐1 cell migration and invasion through CXCL12 secreted by CAFs.

### 3.6 CXCL12/CXCR4 signalling activates the p38/ERK pathway in insulinoma cells

**FIGURE 6:**
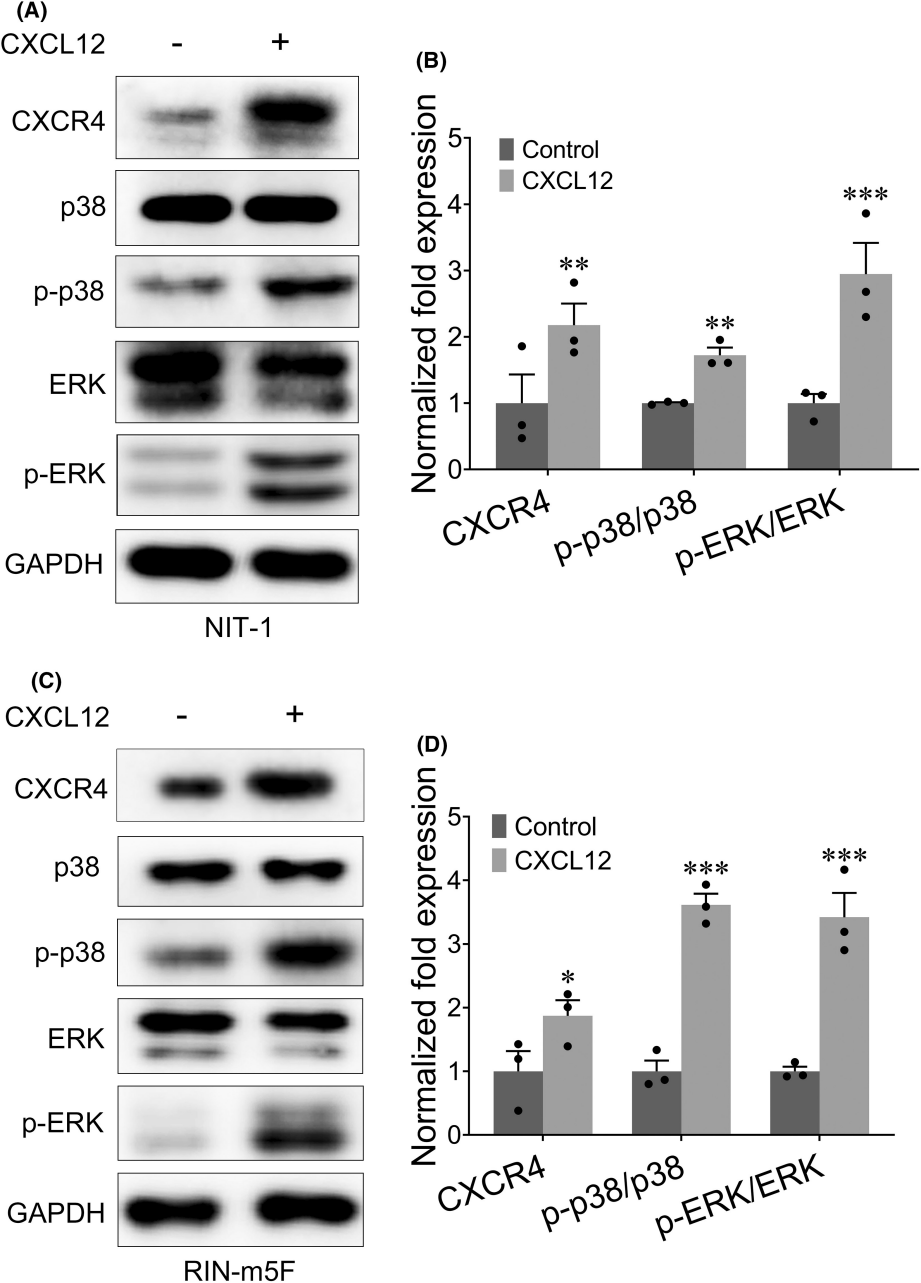
Effect of CXCL12 on the p38/ERK signalling pathway in NIT‐1 and RIN‐m5F cells. (A, B) CXCL12 significantly induced CXCR4, p‐p38 and p‐ERK protein expression in NIT‐1 cells but did not affect p38 and ERK expression. (C, D) CXCL12 significantly increased CXCR4, p‐p38 and p‐ERK protein expression in RIN‐m5F cells but did not affect p38 and ERK expression. * *p <* 0.05; ** *p <* 0.01; *** *p <* 0.001.

CXCL12 can activate the p38/ERK pathway.^30^, ^31^ Therefore, we evaluated the effect of CXCL12 on the p38/ERK pathway in insulinoma cells. We first investigated the expression of CXCR4, the receptor for CXCL12, and found that CXCR4 expression was upregulated in both NIT‐1 cells and RIN‐m5F insulinoma cells. As shown in Figure 6A–D, CXCL12 treatment also resulted in increases in the levels of phospho‐p38 and phospho‐ERK in both NIT‐1 cells and RIN‐m5F insulinoma cells, while p38 and ERK expression did not significantly differ between PBS‐treated and CXCL12‐treated cells. These results show that CXCL12 binds to CXCR4 to increase the levels of p‐p38 and p‐ERK.

## 4 DISCUSSION

**FIGURE 7:**
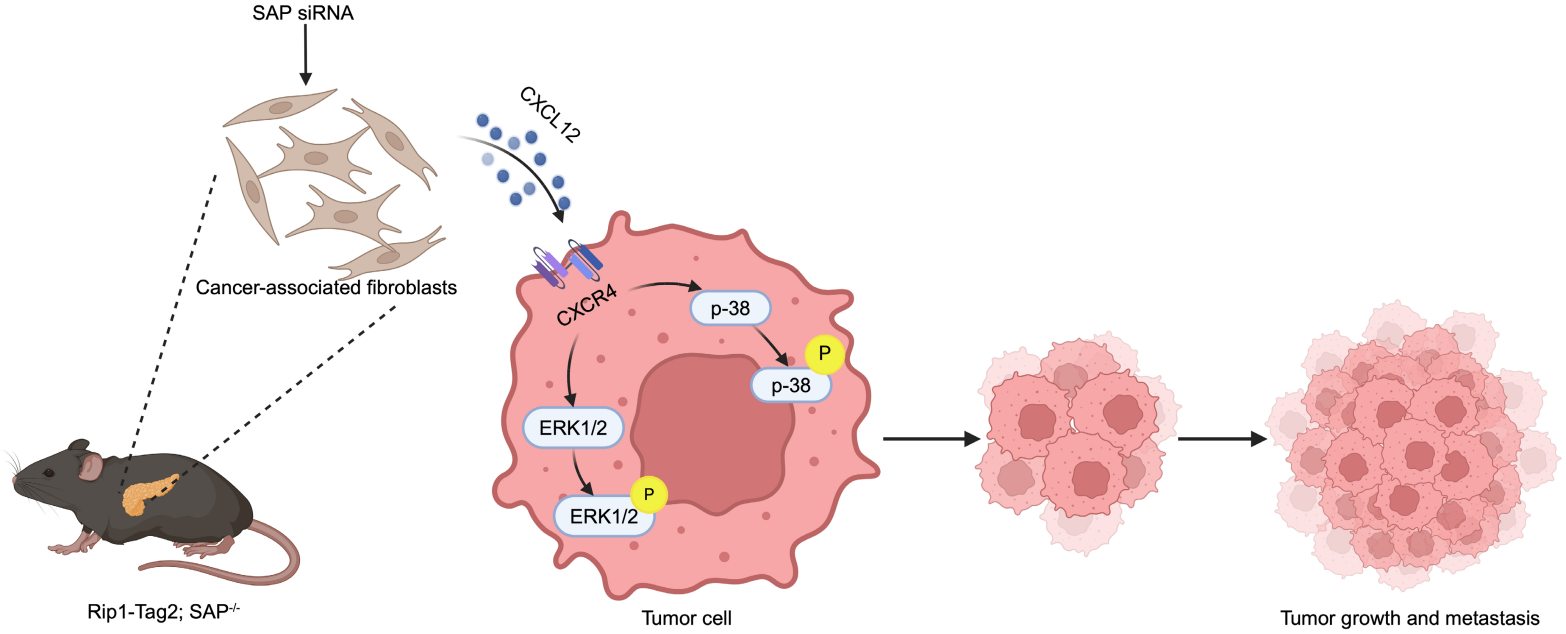
Mechanistic diagram. Schematic diagram of the role of serum amyloid P component (SAP) in insulinoma and the mechanism by which SAP deletion inhibits malignant insulinoma progression.

In the present study, we report the important finding that SAP deletion promotes insulinoma growth and metastasis by upregulating CXCL12, as determined using Rip1‐Tag2 and Rip1‐Tag2;SAP^−/−^ mice. Moreover, SAP deletion promotes CXCL12 secretion by CAFs, thereby increasing NIT‐1 cell migration and invasion. Furthermore, we demonstrate that SAP deletion‐induced upregulation of CXCL12/CXCR4 promotes insulinoma growth and metastasis by activating the p38/ERK signalling pathway (Figure 7).

SAP, a member of the PTX family of proteins, activates the classical complement system and contributes to host defence, tissue clearance and inflammatory regulation.^32^ SAP is associated with amyloidosis and is involved in the regulation of fibrosis.^3^ Previous studies have reported that the serum concentration of SAP in patients with breast cancer and lung cancer is correlated with the progression of these cancers.^33^ However, the role of SAP in insulinoma remains unclear. We crossed Rip1‐Tag2 mice and SAP^−/−^ mice to generate Rip1‐Tag2;SAP^−/−^ mice and found that SAP deletion significantly promoted insulinoma growth, invasion and metastasis by increasing insulinoma cell proliferation. Furthermore, we revealed SAP concentration in the liver tissues of Rip1‐Tag2;SAP^−/−^ mice and found the SAP concentration was very low in the liver tissues of Rip1‐Tag2;SAP^−/−^ mice. Therefore, liver‐produced SAP did not affect insulinoma progression in the Rip1‐Tag2;SAP^−/−^ mice. Insulinomas are the most common functioning pNET and 2.4%–17.9% of insulinomas are malignant. Malignant insulinoma is usually diagnosed in a late stage and approximately 50% of patients already have metastatic disease at the time of diagnosis.^34^ When distant metastasis has occurred, the median survival time is approximately 2 years.^35^ The medical treatment of malignant insulinoma includes surgical resection, chemotherapy and tumour‐targeted radiotherapy.^36^ None of these treatments can cure patients with malignant insulinoma. Therefore, the early diagnosis and timely and rational treatment of malignant insulinoma are greatly needed. To this end, our findings show the promise of SAP as a novel therapeutic agent that may benefit patients with malignant insulinoma.

To investigate how SAP deletion promotes insulinoma growth and metastasis, we performed protein chip assays on the serum of Rip1‐Tag2 and Rip1‐Tag2;SAP^−/−^ mice to screen cytokines known to be associated with inflammatory factors. The results of the protein chip assays and ELISA showed that the concentration of CXCL12 in the serum of Rip1‐Tag2;SAP^−/−^ mice was significantly greater than that in the serum of Rip1‐Tag2 mice. These findings show that SAP deletion increases the CXCL12 concentration, which promotes insulinoma growth and metastasis. It has been reported that CAFs are able to secrete factors such as CXCL12 that promote cancer cell proliferation, cancer cell migration and tumour metastasis.^21^ Therefore, CXCL12 secreted by CAFs can promote tumour progression. We found that SAP deletion promoted the secretion of CXCL12 by CAFs and that CXCL12 promoted insulinoma cell migration and invasion.

The results obtained in the spontaneous insulinoma mouse model suggested that CXCL12 might be involved in the promoting effect of SAP deletion on malignant insulinoma growth and metastasis. The CXCL12/CXCR4 axis has been reported to be a key signalling axis for tumour growth and metastasis through the activation of multiple pathways, such as the p38, ERK1/2 and SAPK/JNK pathways.^37^, ^38^ Therefore, we speculate that CXCL12 may activate the above signalling pathways to play an important role in insulinoma progression through binding to its receptor CXCR4. We observed that CXCL12/CXCR4 signalling increased the p‐p38 and p‐ERK levels in NIT‐1 and RIN‐m5F cells. These findings show that SAP deletion promotes insulinoma progression by increasing the secretion of CXCL12 from CAFs via the p‐p38/p‐ERK pathway.

Taken together, the results of this study demonstrate that SAP deletion is capable of promoting the growth, invasion and metastasis of malignant insulinomas in the Rip1‐Tag2 mouse model of spontaneous insulinoma. The promoting effect of SAP deletion on insulinoma progression was exerted mainly through CXCL12 secreted by CAFs. Further study indicated that CXCL12 promoted the migration and invasion of insulinoma cells by activating the p38/ERK signalling pathway. Although more research is needed to determine the exact molecular mechanisms associated with the promoting effect of SAP deletion on malignant insulinoma progression, SAP may have therapeutic potential for malignant insulinomas.

## AUTHOR CONTRIBUTIONS

**Guangchun Jiang:** Data curation (lead); formal analysis (equal); investigation (equal); methodology (equal); writing – original draft (equal). **Shuo Xu:** Investigation (equal); methodology (equal). **Xiaobin Mai:** Investigation (equal); methodology (equal). **Juan Tu:** Investigation (equal); methodology (equal). **Le Wang:** Investigation (equal); methodology (equal). **Lijing Wang:** Conceptualization (supporting); data curation (equal). **Yaping Zhan:** Supervision (equal); validation (equal). **Yan Wang:** Supervision (equal); validation (equal). **Qianqian Zhang:** Supervision (equal); validation (equal). **Lingyun Zheng:** Supervision (equal); validation (equal). **Jiangchao Li:** Supervision (equal); validation (equal). **Pei Tang:** Funding acquisition (equal); supervision (equal); validation (equal). **Cuiling Qi:** Conceptualization (lead); funding acquisition (equal); methodology (equal); project administration (equal); visualization (equal); writing – original draft (equal); writing – review and editing (equal).

## CONFLICT OF INTEREST STATEMENT

The authors confirm that there are no conflicts of interest.

## Supporting information


**Appendix S1:** Supporting Information.

## Data Availability

The data that support the findings of this study are available upon request from the corresponding author. The data are not publicly available due to privacy or ethical restrictions.
