# Supplementary material for: SAP deletion promotes malignant insulinoma progression by inducing CXCL12 secretion from CAFs via the CXCR4/p38/ERK signalling pathway
Source: J Cell Mol Med. 2024 May 20;28(10):e18397. doi: 10.1111/jcmm.18397 (PMC11103456; doi:10.1111/jcmm.18397)
Supplement: Supplementary file 1 — Appendix S1: Supporting Information. [file JCMM-28-e18397-s001.docx]

**Supporting Information**

**SAP deletion promotes malignant insulinoma progression by inducing CXCL12 secretion from CAFs via the CXCR4/p38/ERK signaling pathway**

Guangchun Jiang^1^ | Shuo Xu^1^ | Xiaobin Mai^1^ | Juan Tu^1^ | Le Wang^1^ | Lijing Wang^1^ | Yaping Zhan^1^ | Yan Wang^1^ | Qianqian Zhang^1^ | Lingyun Zheng^1^ | Jiangchao Li^1^ | Pei Tang ^1^ | Cuiling Qi^1^

^1^School of Basic Medical Sciences, Guangdong Pharmaceutical University, Guangzhou, Guangdong 510006, China

**Figure S1.** SAP expression in patients with insulinoma or mice. Page 2


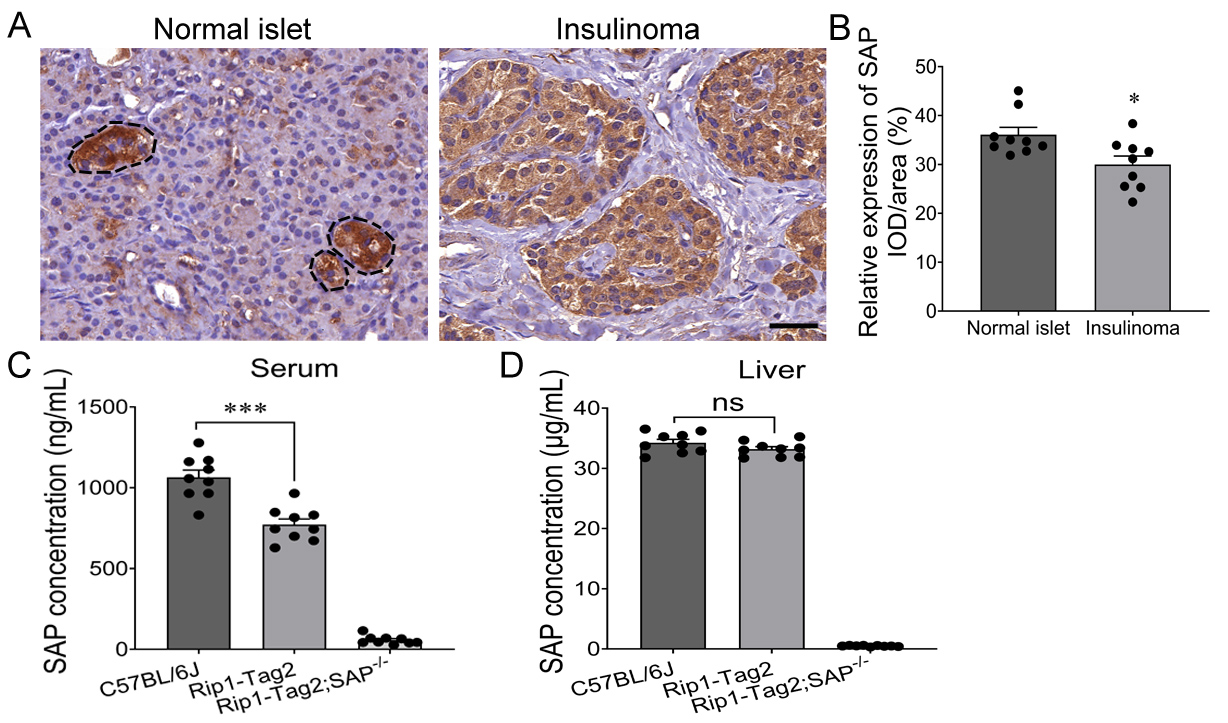


**Supplemental Figure 1** SAP expression in patients with insulinoma or mice. (A) Representative immunohistochemical images of normal islet and insulinoma from patients with malignant insulinoma. (B) Statistical analysis demonstrated that SAP expression was significantly decreased in the insulinoma tissues from the patients with malignant insulinoma compared with that in the normal islets from the paracancerous tissues of the patients with malignant insulinoma. (C) Serum concentrations of SAP in C57BL/6J, Rip1-Tag2 and Rip1-Tag2;SAP^-/-^ mice. (D) Liver homogenate concentrations of SAP in C57BL/6J, Rip1-Tag2 and Rip1-Tag2;SAP^-/-^ mice. ns, no significant difference; * *p* < 0.05; *** *p* < 0.001. Scale bar = 50 µm.
